# Acetaldehyde exposure underlies functional defects in monocytes induced by excessive alcohol consumption

**DOI:** 10.1038/s41598-021-93086-y

**Published:** 2021-07-01

**Authors:** Shunsuke Shiba, Nobuhiro Nakamoto, Po-Sung Chu, Keisuke Ojiro, Nobuhito Taniki, Akihiro Yamaguchi, Rei Morikawa, Tadashi Katayama, Aya Yoshida, Ryo Aoki, Toshiaki Teratani, Takahiro Suzuki, Takeshi Miyamoto, Sachiko Hara, Akira Yokoyama, Takanori Kanai

**Affiliations:** 1 Division of Gastroenterology and Hepatology, Department of Internal Medicine, Keio University School of Medicine, Tokyo, 1608582 Japan; 2 Department of Orthopedic Surgery, Kumamoto University, Kumamoto, Japan; 3 National Hospital Organization Kurihama Medical and Addiction Center, Kanagawa, Japan

**Keywords:** Alcoholic liver disease, Monocytes and macrophages

## Abstract

Increased intestinal permeability and hepatic macrophage activation by endotoxins are involved in alcohol-induced liver injury pathogenesis. Long-term alcohol exposure conversely induces endotoxin immune tolerance; however, the precise mechanism and reversibility are unclear. Seventy-two alcohol-dependent patients with *alcohol dehydrogenase-1B* (*ADH1B*, rs1229984) and *aldehyde dehydrogenase-2* (*ALDH2*, rs671) gene polymorphisms admitted for alcohol abstinence were enrolled. Blood and fecal samples were collected on admission and 4 weeks after alcohol cessation and were sequentially analyzed. Wild-type and ALDH2*2 transgenic mice were used to examine the effect of acetaldehyde exposure on liver immune responses. The productivity of inflammatory cytokines of peripheral CD14^+^ monocytes in response to LPS stimulation was significantly suppressed in alcohol dependent patients on admission relative to that in healthy controls, which was partially restored by alcohol abstinence with little impact on the gut microbiota composition. Notably, immune suppression was associated with *ALDH2/ADH1B* gene polymorphisms, and patients with a combination of ALDH2*1/*2 and ADH1B*2 genotypes, the most acetaldehyde-exposed group, demonstrated a deeply suppressed phenotype, suggesting a direct role of acetaldehyde. In vitro LPS and malondialdehyde-acetaldehyde adducted protein stimulation induced direct cytotoxicity on monocytes derived from healthy controls, and a second LPS stimulation suppressed the inflammatory cytokines production. Consistently, hepatic macrophages of ethanol-administered ALDH2*2 transgenic mice exhibited suppressed inflammatory cytokines production in response to LPS compared to that in wild-type mice, reinforcing the contribution of acetaldehyde to liver macrophage function. These results collectively provide new perspectives on the systemic influence of excessive alcohol consumption based on alcohol-metabolizing enzyme genetic polymorphisms.

## Introduction

Alcohol and its metabolites induce systemic damage such as oxidative stress, hepatocyte injury, and mitochondrial damage. Habitual and excessive alcohol consumption gives rise to alcohol-related liver diseases (ALDs) including steatohepatitis, liver fibrosis, and liver cirrhosis. ALD remains the leading cause of death due to alcohol worldwide.

To date, many studies have tried to elucidate the pathogenesis of ALD from the viewpoint of the gut-liver axis^1,2^. Alcohol consumption results in high intestinal permeability, the overgrowth of gram-negative bacteria in the proximal small intestine and increased circulating endotoxin following bacterial translocation^3^. Endotoxins are pathogen-associated molecular patterns (PAMPs) recognized by Toll-like receptors (TLRs). TLR4 and CD14 are essential components for macrophages/monocytes activation by circulating lipopolysaccharides (LPS)^4^, the major components of the outer membrane of gram-negative bacteria. A major innate immune response of TLR4 binding is NF-kB-mediated activation of pro-inflammatory cytokines such as tumor necrosis factor-alfa (TNF-α) and IL-6^5–8^.

Long-term alcohol consumption has negative effects on host immune functions, which might contribute to suppressed immunity and potential susceptibility to systemic infection^9^. The negative effects of alcohol and its metabolites on monocytes have been studied both in humans and in murine models. These studies have demonstrated the evidence of reduced pro-inflammatory cytokine production in response to LPS stimulation and dysregulated antigen presentation on monocytes owing to chronic alcohol consumption^10–12^; however, the precise mechanism and reversibility with alcohol withdrawal still remain unclarified. Human peripheral blood monocytes have been classified according to their surface expression patterns of CD14 and CD16 into the following three major subsets: classical (CD14^high^CD16^−^), intermediate (CD14^high^CD16^+^), and non-classical (CD14^low^CD16^+^)^13^. These subsets are functionally distinct and play different roles in various diseases^14,15^, however, the contribution of each cell subset to ALD pathogenesis has not been well studied.

Acetaldehyde is a well-known highly toxic metabolite of alcohol. Consumed alcohol is mainly metabolized by alcohol dehydrogenases (ADHs) or cytochrome P450 2E1 (CYP2E1) to acetaldehyde, which is then metabolized to acetate by aldehyde dehydrogenases (ALDHs). In East Asians, the fast-metabolizing form of ADH1B is encoded by ADH1B*2 (rs1229984), affecting more than 90% of the Japanese population^16^. ALDH2 is a major enzyme in acetaldehyde oxidation in humans and its genetic polymorphism (rs671) determines blood or tissue acetaldehyde concentration after alcohol consumption. Individuals with ALDH2 *1/*2 or ALDH2 *2/*2 polymorphisms have much lower activity than those with homozygous ALDH2 *1/*1. Approximately half of the population in East Asia possesses the ALDH2 *2 allele, in contrast to non-Asians, who rarely possess this^17^. ALDH2*2 allele carriers show high acetaldehyde concentrations after alcohol consumption^18^, and extensive DNA damage is induced by chronic excessive drinking^19,20^. Acetaldehyde accumulated in the intestine disrupts the barrier function, which potentially involves the gut–liver axis^21,22^. In addition to the promotion of ethanol-induced gut barrier dysfunction in mice with ALDH2 deficiency^21^, acetaldehyde impairs microtubule-dependent protein trafficking pathways leading to hepatocyte ballooning^23^, results in the formation of immunogenic protein adducts^24^, and increases hepatic stellate cell activation and production of fibrillar collagen^25^. In contrast, a meta-analysis of Asian studies has shown a strong protective effect of ALDH2 deficiency against alcoholic liver cirrhosis as well as alcohol dependence^26^. Furthermore, the results of large surveys of Japanese alcohol-dependent (AD) patients have demonstrated that the inactive ALDH2*1/*2 genotype is associated with a lower risk of alcoholic liver cirrhosis^27,28^. These conflicting findings suggest that acetaldehyde is associated with both fascinating and protective aspects with respect to the development of alcoholic cirrhosis, which prompted us to examine the role of acetaldehyde as an inflammatory mediator, especially in regulating immune response during alcohol exposure.

In the current study, we examined changes in immunological impairment of monocytes in AD subjects following alcohol withdrawal. We unexpectedly noticed that functional impairment of monocytes was closely associated with genetic polymorphisms in alcohol-metabolizing enzymes and thus explored the potential mechanism involved using in vitro assays and murine models.

## Material and methods

### Patients and samples

This study included 72 Japanese male AD patients (average age 52.4 years) who were admitted to the National Hospital Organization Kurihama Medical and Addiction Center for the treatment of alcoholism (Table 1). They fulfilled the following criteria: continued to drink more than 60 g/day of ethanol, did not use alcohol-aversive drugs, were not related to another etiology of liver disease (hepatitis B, C, PBC, and AIH), and had never been admitted for abstinence previously. Blood samples were collected early in the morning, after overnight fasting, on the next day of admission, and at the end of the 4-week of hospital stay. A BD vacutainer cell preparation tube™ was used to isolate peripheral blood monocytes (PBMCs), which were stored at − 80 °C. Thirteen PBMC samples as controls were collected from healthy male donors without drinking habits and were processed. The institutional review board of Keio University School of Medicine and National Hospital Organization Kurihama Medical and Addiction Center approved all human studies (No. 20140211) according to the guidelines of the 1975 Declaration of Helsinki (2008 revision). The study subjects were prospectively recruited, and each subject provided prior written informed consent for blood sampling, study participation, and analysis of clinical data.

**Table 1:** Characteristics of patients with alcohol dependence.

Background characteristics	Mean ± SD			
Age, yrs	52 ± 4			
Cirrhosis (Y/N)	8/64			
Child Pugh score (A/B/C)	5/3/0			
AST, IU/L	86 ± 16			
ALT, IU/L	51 ± 8			
γ-GTP, IU/L	370 ± 61			
Alb, g/dL	4.1 ± 0.6			
T-bil, mg/dL	1.0 ± 0.4			
PT-INR	1.02 ± 0.22			
Type IV collagen, ng/mL	240 ± 20			
FBS, mg/dL	92 ± 10			
WBC, × 10^3^/μL	6.0 ± 0.3			
Hb, g/dL	13.8 ± 0.2			
Plt, × 10^4^/μL	20.3 ± 1.1			
TC, mg/dL	182 ± 28			
HDL-C, mg/dL	58 ± 9			
TG, mg/dL	132 ± 14			

Ethanol (g/day)	n			
0–49	2			
50–99	20			
100–149	32			
150–199	8			
200–249	3			
250–299	3			
300–349	4			

*ALDH2 (Glu487Lys)*	*ADH1B (His47Arg)*			
	*1/*1	*1/*2	*2/*2	Total
*1/*1	14	13	17	44
*1/*2	7	11	10	28
Total	21	24	27	72

### ADH1B and ALDH2 genotypes

The DNA of each subject was extracted from their blood samples using a QIAamp DNA Blood Mini Kit (Qiagen, USA). Polymerase chain reaction–restriction fragment length polymorphism methods were used to analyze lymphocyte DNA samples from all subjects, without knowledge of their status, to determine their *ADH1B* and *ALDH2* genotypes^29^.

### Isolation of human PBMCs

Human PBMCs were isolated by density centrifugation. Human blood was collected into BD Vacutainer CPT (BD, USA) tubes and centrifuged at 470× *g* for 20 min at room temperature. PBMCs were collected at the interphase, washed, and resuspended in FACS buffer.

### Flow cytometric analysis

We performed cell surface staining to characterize the cell populations in each sample. Briefly, cells were incubated with specific fluorescence-labeled monoclonal antibodies at 4 °C for 30 min. CD14 and CD16 were used to identify the monocyte subpopulation. CD3, CD56, BDCA2, and CD123 were used for T cells, NK cells, NKT cells, and plasmacytoid dendritic cells. Events were acquired with a FACS Canto II (Becton Dickinson, USA) and analyzed with FlowJo software v.10.3 (Tree Star Inc., USA) (https://www.flowjo.com/solutions/flowjo). The following antibodies were used for cell surface staining: PE-Cy7 Mouse Anti-Human CD14 (BD Pharmingen), APC-Cy7 Mouse Anti-Human CD16 (BD Pharmingen), FITC Mouse Anti-Human CD3 (BD Pharmingen), PE-Cy7 Mouse Anti-Human CD56 (BD Pharmingen), APC anti-human CD303 (BDCA-2) Antibody (BioLegend), and PerCP/Cyanine5.5 anti-human CD123 Antibody (BioLegend).

### Isolation of CD14^+^ monocytes and cell stimulation

CD14^+^ monocytes were isolated by immunomagnetic positive selection using the EasySep™ Human CD14 Positive Selection Kit (STEMCELL technologies, Canada). Acquired CD14^+^ monocytes were seeded in 24-well plates at 5 × 10^4^ cells/well and stimulated with 5 ng/mL LPS for 24 h at 37 °C.

### RT-qPCR

Total RNA was extracted from cells using TRIzol reagent (Invitrogen, USA), as per the manufacturer’s protocol. Complementary DNA was synthesized by reverse transcription using the iScript™ cDNA Synthesis Kit (Bio-Rad, Hercules, USA). To measure the quantity, real-time PCR was performed using the SYBR green RT-qPCR kit with the predesigned primers. The level of target gene expression was normalized against glyceraldehyde-3-phosphate dehydrogenase (GAPDH) expression in each sample.　The following primers were used for SYBR green assays (BIO-RAD, Japan): *TNF* (qHsaCEP0040184), *IRAK-1* (qHsaCEP0057865), *IRAK-3* (*IRAK-M*; qHsaCIP0031947), *CD274* (*PD-L1*;qHsaCIP0039192), and *PDCD1LG2* (*PD-L2*; qHsaCID0015625).

### Quantification and analysis of cytokines

Cytokine production in cell supernatants was measured using the BD™ Cytometric Bead Array (CBA) Human Th1/Th2/Th17 cytokine kit (BD, USA) and were acquired on the BD FACS Canto™ II flow cytometer. Acquired data were analyzed using FCAP Array™ software v.3.0 (BD, USA) (https://www.bdbiosciences.com/jp/applications/research/bead-based-immunoassays/analysis-software/fcap-array-software-v30/p/652099).

### Fecal sample collection and DNA extraction

Fresh fecal samples were collected using stool collection tubes and an anaerobiosis generator was added to the samples to favor the preservation of anaerobic bacteria at the outpatient clinic of Keio University Hospital. We selected four AD patients who achieved the recovery from diminished cytokines production following alcohol abstinence. The samples were processed immediately and frozen at − 80 °C for bacterial preservation. Bacterial DNA was isolated as described previously^30^. In brief, bacterial DNA was isolated by the enzymatic lysis method using lysozyme (Sigma-Aldrich, USA) and achromopeptidase (Wako). DNA samples were then purified by treating with ribonuclease A (Wako, Japan), followed by precipitation with 20% polyethylene glycol solution (PEG6000 in 2.5 M sodium chloride). DNA was then pelleted by centrifugation, rinsed with 75% ethanol, and dissolved in tris–EDTA buffer.

### 16S rRNA metagenomic analysis

The hypervariable V3–V4 region of the 16S gene was amplified using Ex Taq Hot Start (TAKARA Bio Inc., Japan) and subsequently purified using AMPure XP (Beckman Coulter, USA). Mixed samples were prepared by pooling approximately equal amounts of each amplified DNA sample and sequenced using the Miseq Reagent Kit V3 (600 Cycle) and Miseq sequencer (Illumina, USA), according to the manufacturer’s instructions. Sequences were analyzed using the QIIME2 software package v.2019.10 (https://qiime2.org) ^31,32^. Paired-end sequences were joined using a fastq-join tool in the ea-utils software package (https://doi.org/10.2174/1875036201307010001). High-quality sequences per sample (15,000) were randomly chosen from quality filter-passed sequences. After trimming both primer sequences using cutadapt (https://doi.org/10.14806/ej.17.1.200) followed by chimera detection by the USEARCH de novo method^33^, the sequences were assigned to operational taxonomic units (OTUs) using the UCLUST algorithm^34^ with a sequence identity threshold of 96%. Taxonomic assignments of each OTU were made by similarity searching against the publicly available 16S (RDP version. 10.27 and CORE update 2 September 2012) and NCBI genome database using the GLSEARCH program. Data were rarefied to 10,000 sequences per sample, as determined by the rarefaction curves. Relative abundances of the community members were determined using the rarefied data. UniFrac analysis was performed as described previously^35^. To determine bacterial taxonomy that explained differences between conditions, the linear discriminant analysis effect size method was used^36^.

### Preparation of MAA-Alb and in vitro stimulation

As previous reported, MAA-Alb was prepared by reacting 1.0 mM acetaldehyde and 1.0 mM malondialdehyde with 2 g/L of bovine serum albumin in 0.1 M phosphate buffer containing 2 mM diethylenetriaminepentaacetic acid and 2 mM phytic acid at 37 °C for 3 days and ultrafiltration of the phosphate buffer^37^. CD14^+^ monocytes collected from healthy donors were stimulated with LPS (1 ng/mL), MAA-Alb (10–25 µg/mL), or the combination for 18 h in vitro. In some experiments, CD14^+^ monocytes following the first treatment were further stimulated with 5 ng/mL LPS for 24 h in vitro, followed by cytokine quantification in supernatants.

### Animal experiments

#### Animals and chronic-plus-binge ethanol feeding

We used 6-week-old male C57BL/6 J (wild-type; WT) mice weighing between 17 and 19 g. Mice were obtained from CLEA Japan Inc. The mice were randomly divided into three groups, the control, alcohol group, and withdrawal groups (Fig. 5A), treated differently as follows:

- The control group received the control Lieber-DeCarli diet^38^ ad libitum for 6 weeks. Maltose solution was administered via gavage twice per week.
- The alcohol group received the control Lieber-DeCarli diet ad libitum for the first week and the ethanol Lieber-DeCarli diet containing 5% (v/v) ethanol for the following 6 weeks. Ethanol solution was administered via gavage twice per week.
- The withdrawal group was treated the same as that in the alcohol group for 6 weeks and received the control Lieber-DeCarli diet for the following 3 weeks.

To ensure the same amounts of ethanol intake, each mouse was housed individually and a consistent ethanol Lieber-DeCarli diet containing 5% (v/v) ethanol was provided every day. We prepared a 31.5% (v/v) ethanol solution equivalent to 0.25 g/mL ethanol and 45.0% (w/v) maltose dextrin solution for the gavage. The gavage dose was 20 µL/g body weight, and each mouse received 5 g ethanol/kg body weight or isocaloric maltose. We performed gavage between 7:00 and 9:00, and mice were sacrificed 24 h after the last gavage^38^.

ALDH2*2 transgenic (TG) mice generated as described in prior studies were kindly provided by the Department of Orthopedic Surgery, Keio University School of Medicine^39^. Four WT mice and four TG mice were included in the alcohol group for 6 weeks and their serum and liver were collected and analyzed. All experiments were approved by the regional animal study committees (Keio University, Tokyo, Japan) and were performed in accordance with the ARRIVE guidelines and institutional guidelines and Home Office regulations.

#### Serum transaminase

As a marker of liver injury, serum aspartate aminotransferase and alanine aminotransferase were measured by a simple colorimetric method using the Fuji Dri-Chem 3500™.

#### Intestinal permeabilization in vivo

We prepared 125 mg/mL fluorescein isothiocyanate-dextran (FITC-Dx) solution. After 4 h fasting, mice were gavaged with the FITC-Dx solution at 4 µL/g body weight (500 mg FITC-Dx/kg body weight). Blood samples were collected 4 h after gavage, and serum FITC-Dx levels were measured at an excitation wavelength of 485 nm and emission wavelength of 528 nm.

#### Serum cytokine concentrations

The serum cytokine concentrations were measured using a BD™ Cytometric Bead Array (CBA) Mouse Th1/Th2/Th17 cytokine kit. Data acquisition and analysis were performed as that used for human samples, described previously herein.

#### Intracellular cytokine production and cell population

Mononuclear cells were isolated from the liver and spleen by Percoll density gradient centrifugation. They were seeded in 24-well plates at 5 × 10^5^ cells/well and stimulated with 100 ng/mL LPS for 4 h at 37 °C, in the dark. Before permeabilization, the cells were labeled with CD11b, CD11c, Ly6C, Ly6G, and F4/80. Labeled cells were permeabilized with the BD Cytofix/Cytoperm™ Fixation/Permeabilization Solution Kit, and intracellular staining was performed using Fixable Viability Dye (FVD) eFluor™ 780 for TNF-α. To evaluate the subpopulations of T cells, TCR-β, CD4, CD8, and CD1d-tetramer were separately used for cell surface staining. Prepared samples were acquired with the flow cytometer, BD FACS Canto™ II flow cytometer.

### Statistical analysis

For comparison of two groups, Student’s paired or unpaired *t*-test was used. For comparison of three or more groups, one-way analysis of variance (ANOVA) was used. For categorical variables, the *χ*^2^ test was used. Data are presented as the mean ± SD with *p* < 0.05 considered significant.

## Results

### The number of peripheral CD14^+^CD16^−^ monocytes is decreased in alcohol-dependent patients and partially recovered following alcohol abstinence for 4 weeks

**Figure 1:**
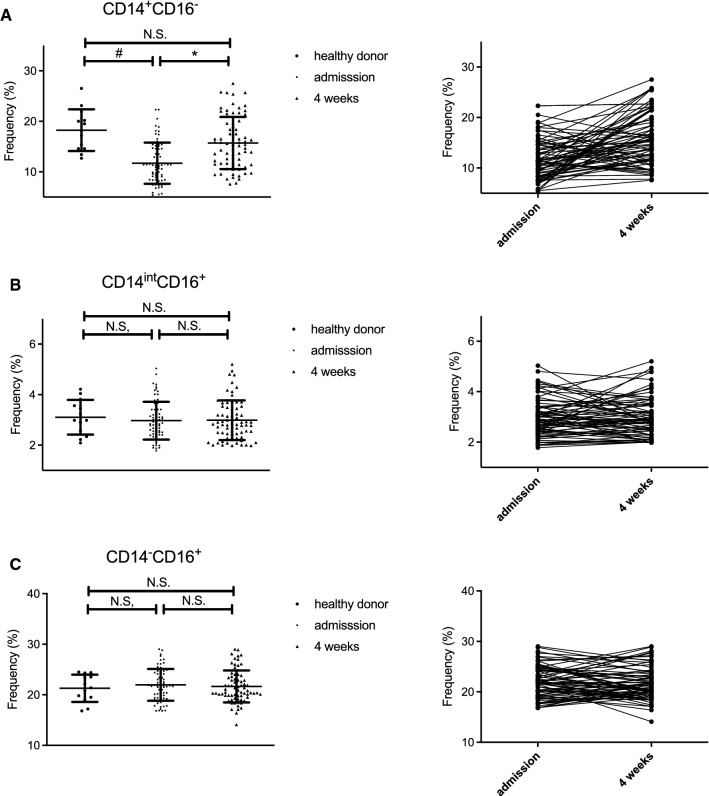
The number of peripheral CD14^+^CD16^−^ monocytes is decreased in alcohol-dependent patients, and partially recovered following alcohol abstinence for 4 weeks. Blood samples were collected from healthy male donors (n = 13) and alcohol dependent patients (n = 72). Left; frequency of CD14^+^CD16^−^ monocytes (**A**), CD14^int^CD16^+^ monocytes (**B**), and CD14^−^CD16^+^ monocytes (**C**) in the indicated groups. Data represent the mean ± SD. ^#^*p* < 0.05 by Student’s unpaired t-test. Right; individual change in the frequency of CD14^high^CD16^−^ monocytes (**A**), CD14^high^CD16^+^ monocytes (**B**), and CD14^low^CD16^+^ monocytes (**C**) following abstinence. ^*^p < 0.05 Student’s paired t-test (admission *vs* 4 weeks).

We obtained blood samples from AD patients both at admission and at 4 weeks after abstinence and compared them with those of healthy controls. The clinical characteristics, alcohol intake, and genetic polymorphisms in the alcohol-metabolizing enzymes of patients and controls are shown in Table 1. PBMCs were isolated and the surface expression of CD14 and CD16 was analyzed by flow cytometry (Supplementary Fig. 1A). The frequency of CD14^+^CD16^−^ monocytes in the PBMCs of AD patients at the time of admission was significantly lower than that in healthy donors and was recovered following alcohol abstinence for 4 weeks (Fig. 1A). However, the difference between AD patients and healthy controls was not observed for other immune cells including CD14^int^ CD16^+^ monocytes (Fig. 1B), CD14^−^CD16^+^ monocytes (Fig. 1C), CD123^+^BDCA2^+^ plasmacytoid DCs, CD3^+^CD56^−^ T cells, CD3^−^CD56^+^ NK cells, and CD3^+^CD56^+^ NKT cells (Supplementary Fig. 1B–G).

### Peripheral CD14^+^CD16^−^ monocytes from alcohol-dependent patients produce less TNF-α and IL-6 after in vitro stimulation with LPS, and this defect is partially recovered following alcohol abstinence for 4 weeks.

**Figure 2:**
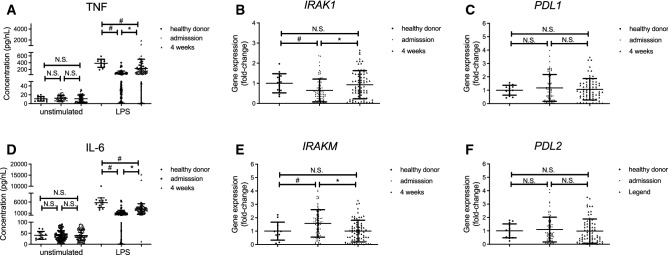
Peripheral CD14^+^CD16^−^ monocytes from alcohol-dependent patients produce less TNF-α and IL-6 after in vitro stimulation with LPS, and the defect is partially recovered following alcohol abstinence for 4 weeks. (**A**,**B**) Concentration of TNF-α (**A**) and IL-6 (**B**) in the culture supernatants of CD14^+^ monocytes isolated from each individual in the indicated groups stimulated with medium or LPS for 24 h in vitro. Data represent the mean ± SD. (**C**–**F**) Gene expression of (**C**) *IRAK-1*, (**D**) *IRAK-M*, (**E**) *PD-L1*, and (**F**) *PD-L2* in peripheral blood mononuclear cells (PBMCs) isolated from each individual in the indicated groups. Data represent the mean ± SD. ^#^*p* < 0.05 by Student’s unpaired t-test. N.S.: not significant.

Given that CD14^+^CD16^−^ monocytes were the main cell subset for which numbers were affected by alcohol abuse, we examined the functional profile of these cells. Sorted peripheral CD14^+^ monocytes from ADs and healthy controls produced low amounts of the inflammatory cytokines, TNF (Fig. 2A) and IL-6 (Fig. 2B) without stimulation. In contrast, after in vitro stimulation with LPS for 24 h, which induced robust inflammatory cytokines production, CD14^+^ monocytes from AD patients produced significantly lower amounts of these inflammatory cytokines compared to those from healthy controls (Fig. 2A,B). Importantly, the potential to produce inflammatory cytokines after LPS stimulation was partially recovered following alcohol abstinence for 4 weeks (Fig. 2A,B). CD14^+^ monocytes from AD patients expressed lower IRAK-1 and higher IRAK-M levels at the time of admission, but their levels were recovered to a similar level as those of healthy donors (Fig. 2C,D), whereas the expression of PD-L1 and PD-L2 was not different between healthy controls and ADs (Fig. 2E,F). Of note, both cytokine production in response to LPS stimulation and the recovery following alcohol abstinence were not affected by any clinical parameter such as age, average ethanol intake before admission, serum transaminase, and type IV collagen level on admission (Supplementary Fig. 2). We initially hypothesized that the altered composition of gut microbiota mediated by alcohol abstinence affected the recovery of monocyte function; however, metagenomics analysis of fecal samples from four AD patients whose monocytes demonstrated a substantial recovery from diminished cytokine production suggested that short-term alcoholic abstinence did not result in a remarkable change in the gut microbiota composition (Supplementary Fig. 3).

### CD14^+^CD16^−^ monocytes in AD patients with ADH1B*2 and ALDH2*1/*2 genotypes demonstrate a deeply suppressed phenotype

**Figure 3:**
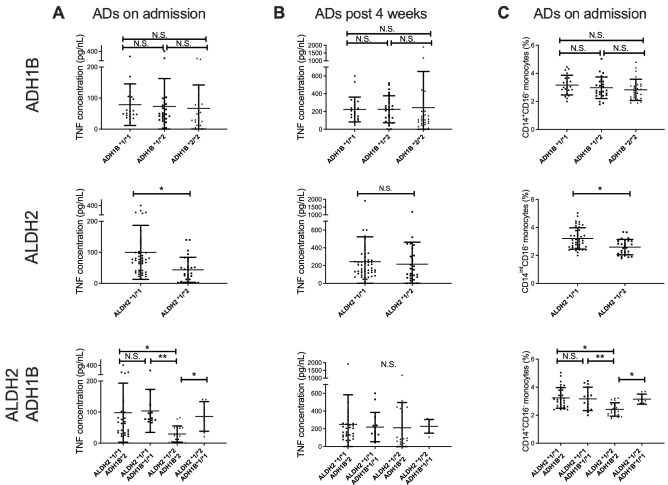
CD14^+^CD16^−^ monocytes in alcohol-dependent (AD) patients with ADH1B*2 and ALDH2*1/*2 genotypes demonstrate a deeply suppressed phenotype. (**A**–**C**) Concentration of TNF-α in the culture supernatants of CD14^+^ monocytes isolated from AD patients on admission (**A**) and at 4 weeks post-abstinence (**B**) according to the genetic polymorphism of *ADH1B* (upper), *ALDH2* (middle), and the combination (lower). (**C**) Frequency of CD14^+^CD16^−^ monocytes isolated from AD patients on admission according to the genetic polymorphism in ADH1B (upper), ALDH2 (middle), and the combination (lower). Data represent the mean ± SD. *p < 0.05 by Student’s paired t-test for two groups or by one-way ANOVA for three or four groups. N.S.: not significant.

As both ethanol and its metabolite acetaldehyde have been reported to alter the intestinal permeability, in addition to their direct cytotoxic effect^21^, we hypothesized that genetic factors of alcoholic metabolism might affect the function of these monocytes. For this, patients were classified according to *ADH1B* or *ALDH2* genotypes and reanalyzed. The ability of CD14^+^ monocytes to produce inflammatory cytokines in response to LPS stimulation did not differ by *ADH1B* polymorphism; however, we noticed that CD14^+^ monocytes from AD patients with the ALDH2*1/*2 genotype produced significantly lower amounts of TNF-α compared to those in AD patients with ALDH2*1/*1 on admission (Fig. 3A). Furthermore, CD14^+^ monocytes from AD patients who carried the combination of ALDH2*1/*2 and ADH1B*2 genotypes, the most acetaldehyde-exposed group, produced the lowest amount of TNF-α in response to LPS stimulation compared to that in patients with the three other genotypic patterns on admission (Fig. 3A). The difference was abolished following alcohol abstinence for 4 weeks (Fig. 3B). We also confirmed that the frequency of CD14^+^ monocytes was also affected by the combination of the alcohol-metabolizing genes (Fig. 3C). Of note, the daily ethanol consumption of ADs before admission did not differ according to the genetic polymorphisms (Supplementary Fig. 4A). These results collectively suggest that functional impairment in the monocytes of AD patients might be regulated by acetaldehyde and not by the simple amount of alcohol consumption. Regardless of the functional difference in monocytes according to the combination of alcohol-metabolizing genes, clinical parameters including serum transaminase levels, and fibrosis markers were comparable between the groups (Table 2 and Supplementary Fig. 4B–G).

**Table 2:** Characteristics according to the genetic polymorphisms of alcohol metabolizing enzymes.

Background characteristics	Mean ± SD	Mean ± SD	Mean ± SD	Mean ± SD	*P*-value^a^
	ADH1B*2 ALDH2*1/*1	ADH1B*1/*1 ALDH2*1/*1	ADH1B*2 ALDH2*1/*2	ADH1B*1/*1 ALDH2*1/*2	
Number of subjects	30	14	21	7	–
Age, yrs	49 ± 5	53 ± 5	53 ± 4	51 ± 7	0.292
Cirrhosis (Y/N)	2/28	2/12	3/18	1/6	–
Child Pugh score (A/B/C)	2/0/0	1/1/0	1/2/0	1/0/0	–
AST, IU/L	90 ± 35	87 ± 26	79 ± 16	85 ± 24	0.278
ALT, IU/L	56 ± 17	47 ± 10	46 ± 8	50 ± 11	0.450
γ-GTP, IU/L	381 ± 79	369 ± 61	357 ± 59	403 ± 98	0.502
Alb, g/dL	4.2 ± 0.5	3.9 ± 1.1	3.9 ± 0.9	4.0 ± 1.4	0.523
T-bil, mg/dL	0.8 ± 0.2	0.7 ± 0.4	1.1 ± 0.6	1.3 ± 0.8	0.413
PT-INR	1.03 ± 0.36	0.91 ± 0.42	1.15 ± 0.30	0.96 ± 0.37	0.324
Type IV collagen, ng/mL	237 ± 39	234 ± 33	250 ± 41	232 ± 47	0.354
FBS, mg/dL	100 ± 6	90 ± 10	84 ± 7	96 ± 9	0.372
WBC, × 10^3^/μL	6.1 ± 0.4	6.6 ± 0.6	5.5 ± 0.5	5.7 ± 0.8	0.405
Hb, g/dL	14.2 ± 0.4	13.7 ± 0.5	13.2 ± 0.5	13.6 ± 0.7	0.561
Plt, × 10^4^/μL	20.8 ± 1.7	21.3 ± 2.9	19.1 ± 2.2	20.4 ± 3.2	0.412
TC, mg/dL	177 ± 30	187 ± 38	180 ± 29	191 ± 39	0.333
HDL-C, mg/dL	61 ± 9	56 ± 11	59 ± 13	56 ± 14	0.420
TG, mg/dL	134 ± 19	127 ± 21	135 ± 17	131 ± 19	0.303

^a^Homogeneity among the four groups based on one-way analysis of variance (ANOVA).

### MAA-Alb enhances LPS stimulation of CD14^+^ monocytes, and cells exposed to MAA-Alb produce less TNF-α upon secondary LPS stimulation

**Figure 4:**
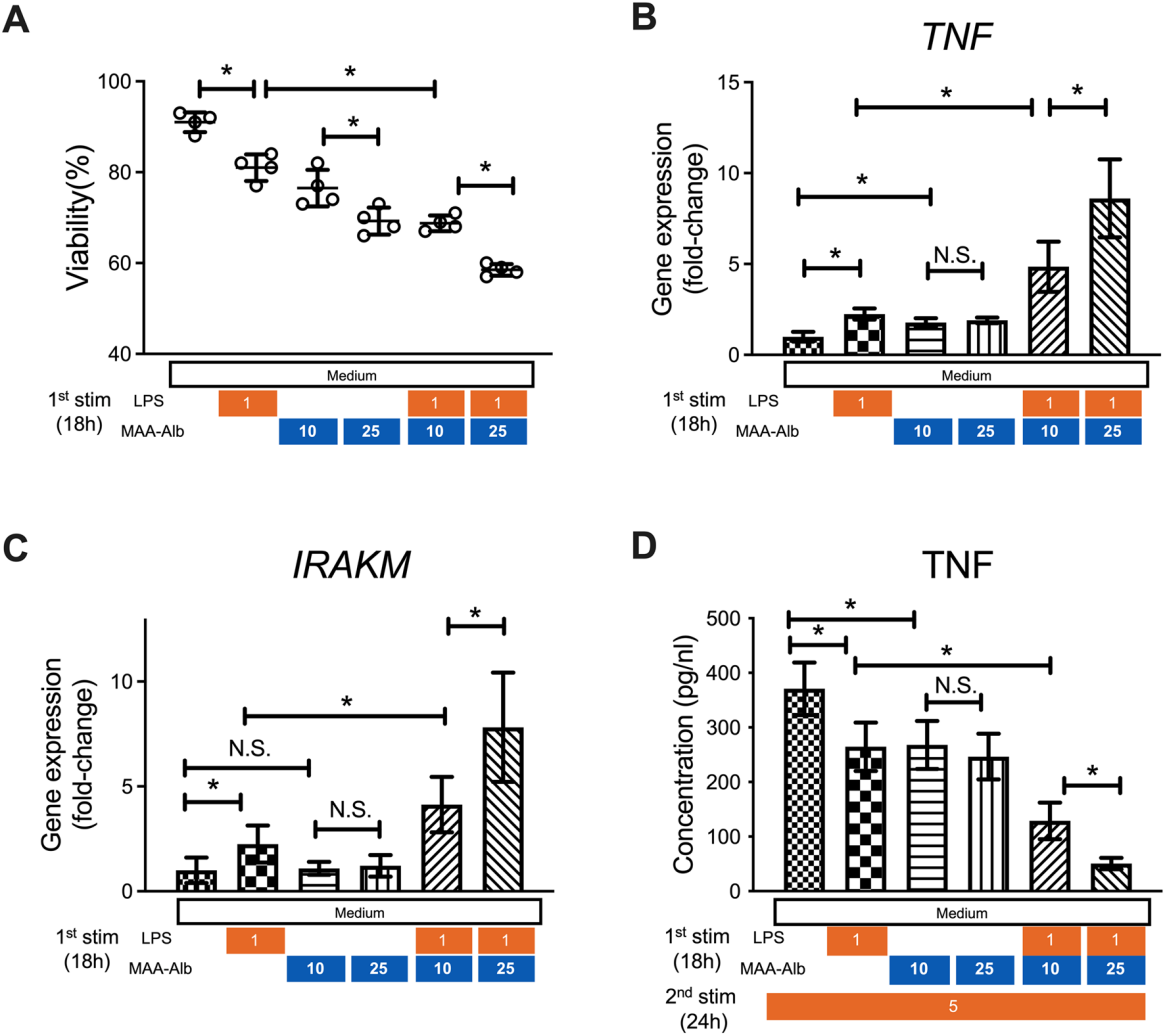
MAA-Alb enhances LPS stimulation of CD14^+^ monocytes, and cells exposed to MAA-Alb produce less TNF-α upon secondary LPS stimulation. CD14^+^ monocytes collected from healthy donors were stimulated in the presence of LPS (1 ng/mL) and Alb (10–25 µg/mL) or MAA-Alb (10–25 µg/mL) for 18 h in vitro. (**A**–**C**) Viability of (**A**) and gene expression of *TNF-α* (**B**) and *IRAK-M* (**C**) in monocytes following stimulation. Cells were stimulated with 5 ng/mL LPS for 24 h. (**D**) Concentration of TNF-α in the culture supernatants of the monocytes following the second LPS stimulation for an additional 24 h in vitro. Data represent the mean ± SD. *p < 0.05, as determined by one-way ANOVA, N.S.: not significant.

CD14^+^ monocytes from the blood of healthy donors were obtained and stimulated with LPS in the presence of acetaldehyde in vitro to examine the direct effect of acetaldehyde on the function of peripheral monocytes. CD14^+^ monocytes demonstrated lower viability after incubation with LPS and MAA-Alb in a dose-dependent manner compared to that in cells without MAA-Alb (Fig. 4A). Monocytes incubated with LPS and MAA-Alb showed higher expression of TNF-α and IRAK-M than those stimulated with LPS alone (Fig. 4B,C). Conversely, monocytes incubated with MAA-Alb and LPS produced lower amounts of TNF-α following secondary LPS stimulation (Fig. 4D). These results collectively reinforce the direct contribution of acetaldehyde to the immune response of monocytes upon continuous LPS stimulation.

### Continuous alcohol administration to mice results in suppressed production of inflammatory cytokines from CD11b^+^ macrophages in the liver

**Figure 5:**
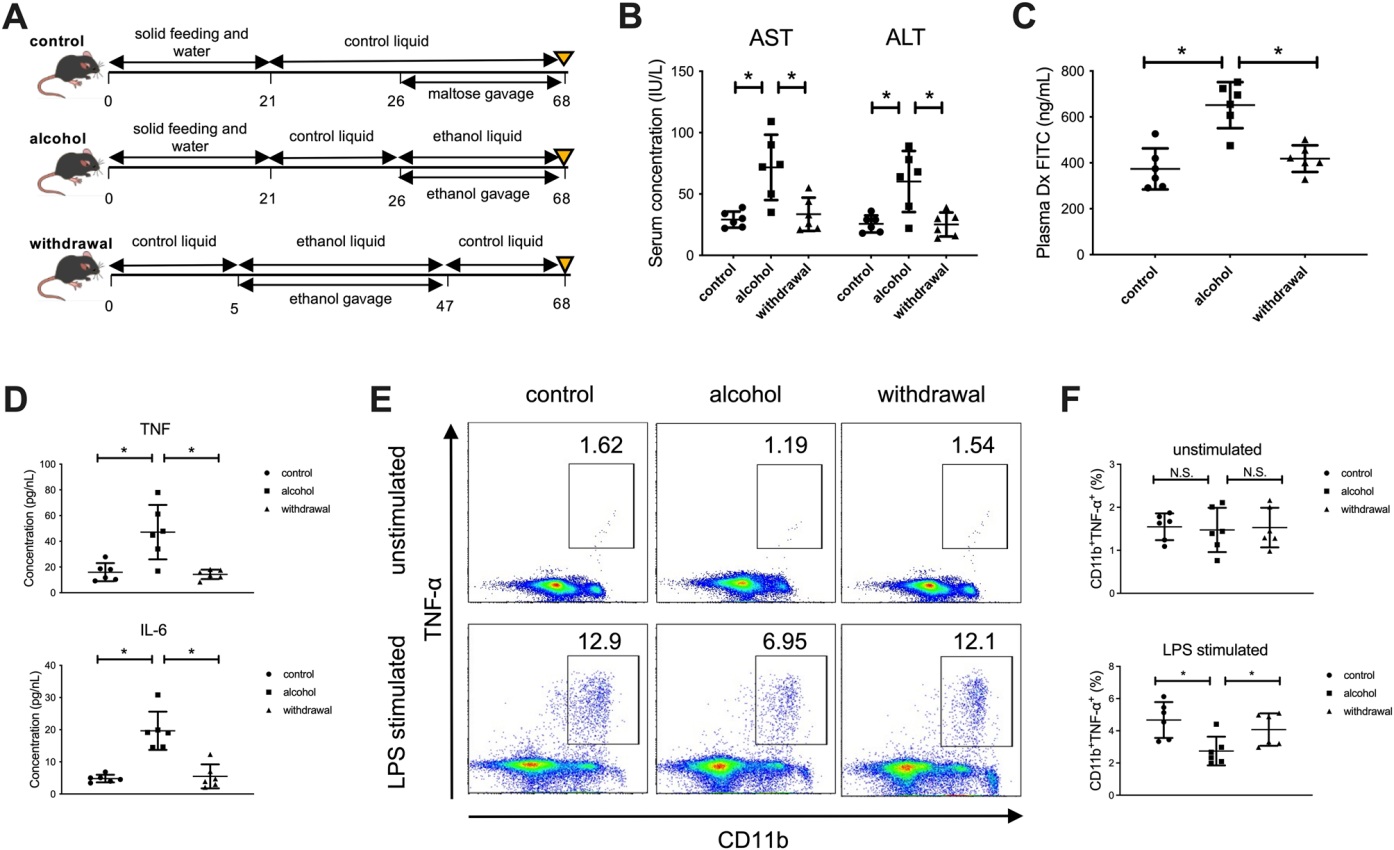
Increased intestinal permeability and the subsequent influx of PAMPs mediated by continuous alcohol administration to mice result in suppressed production of inflammatory cytokines from CD11b^+^ macrophages in the liver. (**A**) Study design. Control mice (n = 6) received a control liquid diet for 26 days. They were gavaged with isocaloric maltose dextrin twice per week for the last 6 weeks. Alcohol-treated mice (n = 6) received the control liquid diet for 5 days and the 5% ethanol liquid diet for 6 weeks. Withdrawal-group mice (n = 6) received the control liquid diet for 5 days and 5% ethanol liquid diet for 6 weeks, followed by feeding with the control liquid diet for 3 weeks as a withdrawal period. (**B**) Serum aspartate aminotransferase (AST) and alanine aminotransferase (ALT) levels. (**C**) Intestinal permeability evaluated by measuring the amount of fluorescence from dextran-FITC (Dx FITC) at 4 h after gavage. (**D**) Serum levels of TNF-α and IL-6. (**E**) Representative surface CD11b and intracellular TNF-α staining of liver mononuclear cells derived from the indicated mice, either unstimulated (upper) or stimulated with LPS for 4 h (lower). (**F**) Frequency of hepatic TNF-α producing CD11b^+^ macrophages from the indicated mice either unstimulated (upper) or stimulated with LPS for 4 h (lower). Data represent the mean ± SD. *p value < 0.05 by one-way ANOVA. N.S.: not significant. Data are representative from three independent experiments.

**Figure 6:**
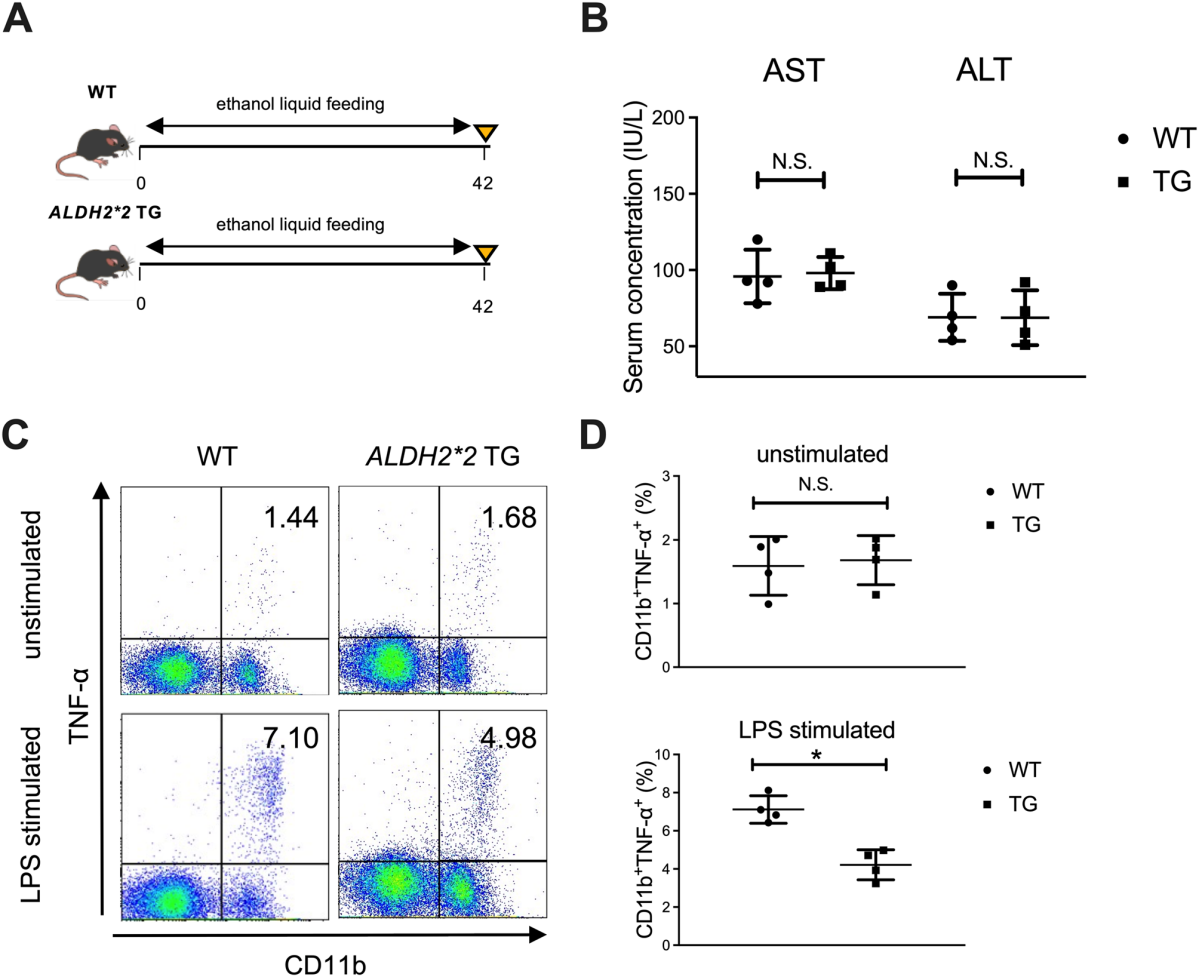
Productivity of inflammatory cytokines from hepatic macrophages in response to LPS stimulation is significantly suppressed in ALDH2*2 transgenic (TG) mice chronically fed ethanol. (**A**) Study design. WT mice (n = 4) or ALDH2*2 TG mice (n = 4) received a control liquid diet for 5 days and 5% ethanol liquid diet for 6 weeks. (**B**) Serum aspartate aminotransferase (AST) and alanine aminotransferase (ALT) levels. (**C**) Representative surface CD11b and intracellular TNF-α staining of liver mononuclear cells derived from the indicated mice either unstimulated (upper) or stimulated with LPS for 4 h (lower). (**D**) Frequency of hepatic TNF-α producing CD11b^+^ macrophages from the indicated mice either unstimulated (upper) or stimulated with LPS for 4 h (lower). Data represent the mean ± SD. *p value < 0.05 by Student’s paired t-test. N.S.: not significant. Data are representative of two independent experiments.

We finally used murine models to examine the effect of continuous alcohol administration and abstinence on the function of macrophages in the liver. Mice were divided into the following three groups: alcohol group, orally administered ethanol gavage for 42 days; withdrawal group, orally administered ethanol gavage for 42 days followed by control liquid feeding for 21 days; control group (Fig. 5A). As expected, alcohol-group mice developed mild liver injury both serologically and histologically, and alcohol abstinence for 3 weeks recovered the damage to the control level (Fig. 5B). Importantly, intestinal permeability, assessed by FITC dextran administration and the level of inflammatory cytokines in the serum, was significantly increased by continuous alcohol administration and was recovered by alcohol abstinence (Fig. 5C,D). Conversely, the production of TNF-α by hepatic CD11b^+^ macrophages after in vitro LPS stimulation was significantly decreased in the alcohol group and was partially recovered in the withdrawal group, consistent with the results shown in human subjects (Fig. 5E,F). Finally, we used ALDH2*2 transgenic mice to examine the direct contribution of alcohol-metabolizing enzymes to the function of liver macrophages in response to LPS stimulation (Fig. 6A). Although serum transaminase levels were comparable to those of WT mice (Fig. 6B), hepatic CD11b^+^ macrophages of ALDH2*2 transgenic mice chronically fed ethanol showed significantly lower production of TNF-α in response to in vitro LPS stimulation than those from WT mice (Fig. 6C,D).

## Discussion

In this study, we showed that chronic alcohol consumption induces hypo-reactivity of peripheral monocytes to LPS in Japanese male AD patients, and short-term abstinence partially restored this reactivity. Furthermore, we obtained similar results with a murine model of chronic alcohol feeding and alcohol withdrawal. Notably, we demonstrated that the immunological defects in the peripheral monocytes of AD patients are partially regulated by genetic polymorphisms in alcohol-metabolizing enzymes and that the most acetaldehyde-exposed patients carrying the combination of ALDH2*1/*2 and ADH1B*2 showed the most impaired function of peripheral monocytes regardless the similar amounts of alcohol intake.

Clinical observations and experimental data have revealed that excessive alcohol use has significant inhibitory effects on the immune system. From the viewpoint of the gut–liver axis, habitual ethanol consumption induces altered composition of the gut microbiota, termed dysbiosis, and subsequent increased intestinal permeability^40^. This results in elevated LPS levels in circulation and subsequent activation of NF-κβ-mediated transcription of proinflammatory cytokines as the first line of defense against foreign bacteria or metabolites in the liver^41^. In contrast, continuous exposure to bacteria and the subsequent immune active state give rise to an immune paralytic state with a higher susceptibility to infection in a specific condition of ALD^9^. In the current study, the number of CD14^+^ CD16^–^ monocytes was significantly decreased in the PBMCs of AD patients, whereas that of other subsets was not affected. Furthermore, these cells were functionally impaired as the production of TNF-α and IL-6 in response to LPS was significantly decreased compared to that in cells of healthy controls. As a potential mechanism of immunological impairment, previous studies have shown that the IL-1-receptor-associated kinase (IRAK) family plays important roles in ALD pathogenesis and the compromised status of alcoholism. In the immune response via TLR signaling, IRAK-M, also known as IRAK-3, inhibits inflammatory cytokine production to prevent excessive inflammation and tissue damage^42^. At the early stage of LPS stimulation to monocytes, the expression of IRAK-1 is predominant and the production of inflammatory cytokines is promoted; however, the expression of IRAK-M gradually becomes dominant and the cytokine productivity per monocyte is decreased, termed LPS tolerance^43,44^. A previous study has demonstrated that IRAK-M-deficient mice show enhanced intestinal permeability, higher serum endotoxin levels, and worse liver injury after ethanol administration, suggesting that IRAK-M negatively regulates the innate immune response with alcoholic liver injuries. In our study, CD14^+^ monocytes derived from AD patients expressed higher levels of IRAK-M and produced fewer inflammatory cytokines in response to LPS than those from healthy controls. These results suggest that macrophages and monocytes in AD individuals are in a state of LPS tolerance, and this condition is related to the insufficient production of inflammatory cytokines, leading to the relief of excessive inflammation in the liver, while contributing to an infectious state. Recent papers have reported the contribution of immune-inhibitory receptors, such as PD-1 and TIM-3, to the immunological defects in patients with acute alcoholic hepatitis^45^; however, the gene expression of PD-L1 was not affected in this study, possibly due to the different state of ALD.

Since alcohol abstinence is still one of the major therapeutic options for ALD, it is critical to determine whether alcohol withdrawal could restore the immunological impairment. We confirmed that the impaired function of inflammatory cytokine production in response to LPS stimulation of circulating monocytes was reversible following short-term abstinence for 4 weeks. As mentioned, gut microbiota affects the intestinal barrier and subsequent immune responses both during the initiation and progression of ALD^46^. Thus, we initially hypothesized that the altered composition of gut microbiota induced by long-term alcohol consumption could be recovered by alcohol withdrawal, leading to improvements in the impaired immune responses. However, the individual changes following short-term abstinence were not evident in our small cohort.

Rather, it is possible that ethanol or its metabolites directly affect the immunological function of monocytes via TLR signaling. Regarding this point, we noticed that the production of inflammatory cytokines by peripheral monocytes in response to LPS stimulation differed according to the combination of *ALDH2/ADH1B* gene polymorphisms in AD patients. Interestingly, we confirmed that AD patients with the ALDH2 *1/*2 and ADH1B*2 genotype combination, affecting approximately 30% of the Japanese population^16^, showed the lowest number of and most impaired cytokine production by peripheral CD14^+^ monocytes. A recent report demonstrated that the number of circulating monocytes is elevated in AD individuals compared to that in healthy controls^47^. Considering that the majority of patients included in this study were Caucasian, patient characteristics based on different alcohol metabolism-related genes and the extent of alcohol consumption might explain the conflicting result. The ALDH2*1/*2 genotype is a major determinant of high blood acetaldehyde exposure after alcohol intake. Although an ADH1B*2 genotype has little effect on blood acetaldehyde levels after alcohol challenge tests using moderate doses of ethanol in non-AD patients^48^, previous studies have suggested that blood acetaldehyde exposure is the highest in drinkers with the ALDH2*1/*2 and ADH1B*2 genotype combination for the following reasons: the slope of the increase in blood acetaldehyde levels according to the increase in blood ethanol levels were found to be steepest for intoxicated AD individuals belonging to this group^49^, the highest levels of N2-ethylidene-dG, an acetaldehyde-DNA adduct, were detected in the leukocytes of AD individuals belonging to this group^19^, and the most severe macrocytic anemia and leukocytopenia^50^ and the slowest recoveries after the cessation of drinking^51^ were observed among AD individuals belonging to this group, suggesting the strongest bone marrow suppression as a result of the highest blood acetaldehyde exposure. In contrast, the direct effect of acetaldehyde on the function of immune cells has not been elucidated to data. We confirmed that peripheral monocytes of healthy controls stimulated with LPS and MAA produced lower amounts of TNF-α in response to the second LPS stimulation in parallel with the upregulation of *IRAK-M* gene expression. Although we did not examine the immunological aspect in human livers, we demonstrated that hepatic CD11b^+^ macrophages of ALDH2*2 transgenic mice chronically fed ethanol were functionally impaired in response to LPS stimulation, similar to the results in humans. Of note, another study demonstrated that patients with ALDH2*1/*2 or *Aldh2-*deficient mice had much higher levels of blood acetaldehyde and glucocorticoids, leading to immune suppression and the attenuated liver injury by inhibiting T-cell glucose metabolism^52^. These findings together might explain the regulated liver injuries regardless of continuous exposure to a high concentration of acetaldehyde following alcohol consumption in this specific population, as reported in large cross-sectional studies of Japanese AD patients^27,28^.

Collectively, our findings suggest a previously unrevealed mechanism of how chronic excessive alcohol intake induces immunological tolerance of monocytes. Strikingly, acetaldehyde directly affects the immune response, which is reversible, following alcohol abstinence. Although further investigations with a large cohort validation are needed, the results of this study provide a new perspective on the systemic influence of excessive alcohol consumption according to the genetic polymorphisms of alcohol-metabolizing enzymes in Japanese AD patients.

## Supplementary Information

Supplementary Information.
