# Supplementary material for: Acetaldehyde exposure underlies functional defects in monocytes induced by excessive alcohol consumption
Source: Sci Rep. 2021 Jul 1;11:13690. doi: 10.1038/s41598-021-93086-y (PMC8249592; doi:10.1038/s41598-021-93086-y)
Supplement: Supplementary file 1 — Supplementary Information. [file 41598_2021_93086_MOESM1_ESM.pdf]

SUPPORTING MATERIALS

Acetaldehyde exposure underlies functional defects in monocytes induced by excessive alcohol consumption

Shunsuke Shiba, Nobuhiro Nakamoto, Po-Sung Chu, Keisuke Ojiro, Nobuhito Taniki, Akihiro Yamaguchi, Rei Morikawa, Tadashi Katayama, Aya Yoshida, Ryo Aoki, Toshiaki Teratani, Takahiro Suzuki, Takeshi Miyamoto, Sachiko Hara, Akira Yokoyama, Takanori Kanai

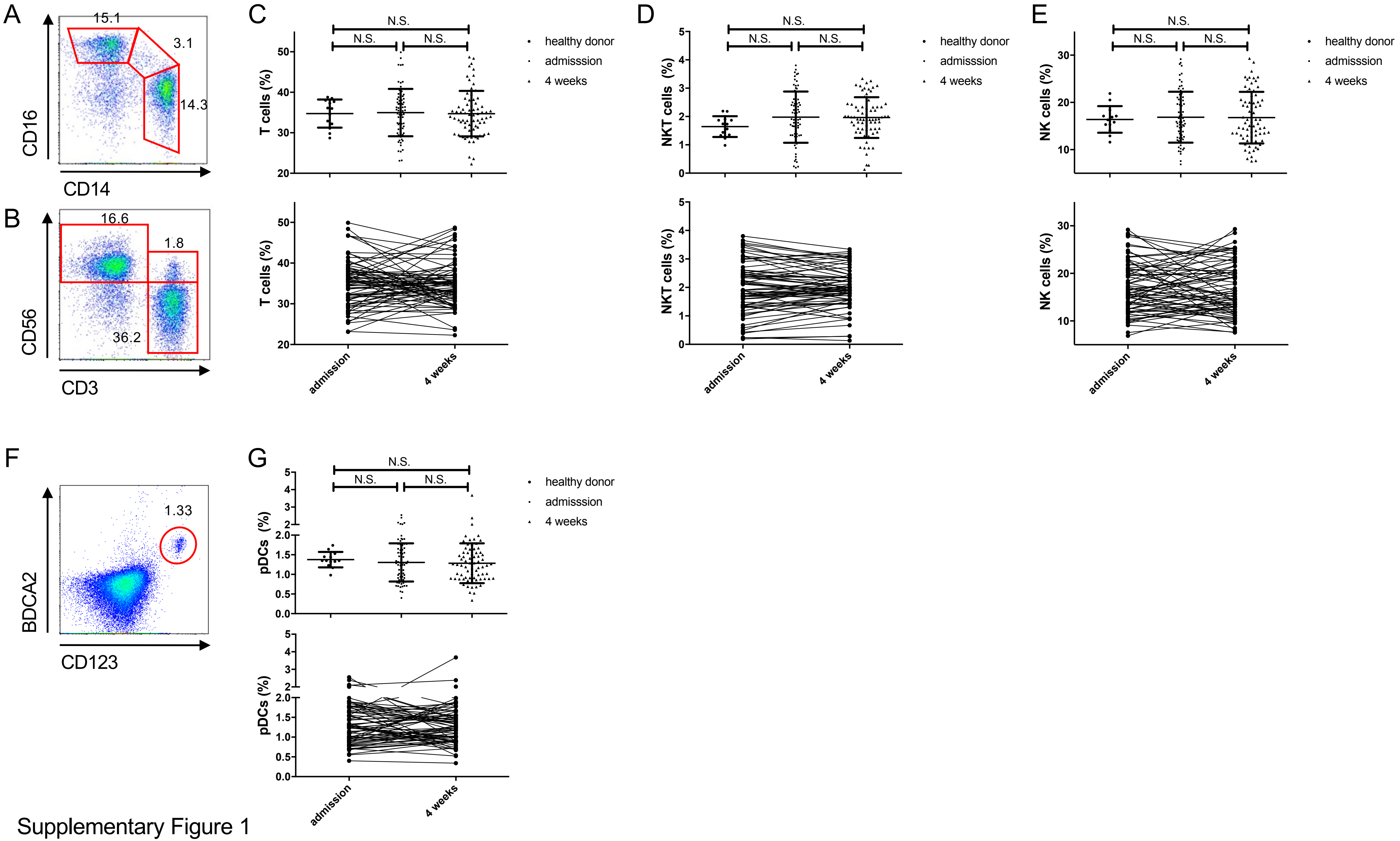

Supplementary Figure 1

(A-B) Representative CD14 and CD16 staining (A) and CD3 and CD56 staining (B) of peripheral blood cells. (C-E) Upper; frequency of peripheral $CD3^{+}CD56^{-}$ T cells (C), $CD3^{+}CD56^{+}$ NKT cells (D), and $CD3^{-}CD56^{+}$ NK cells (E) of the indicated group. Lower; Individual change of the frequency of each cell subset in AD patients following alcohol abstinence. (F) Representative CD123 and BDCA2 staining of peripheral blood cells. (G) Upper; frequency of peripheral $CD123^{+}BDCA2^{+}$ pDCs of the indicated group. Lower; Individual change of the frequency of pDCs in AD patients following alcohol abstinence.

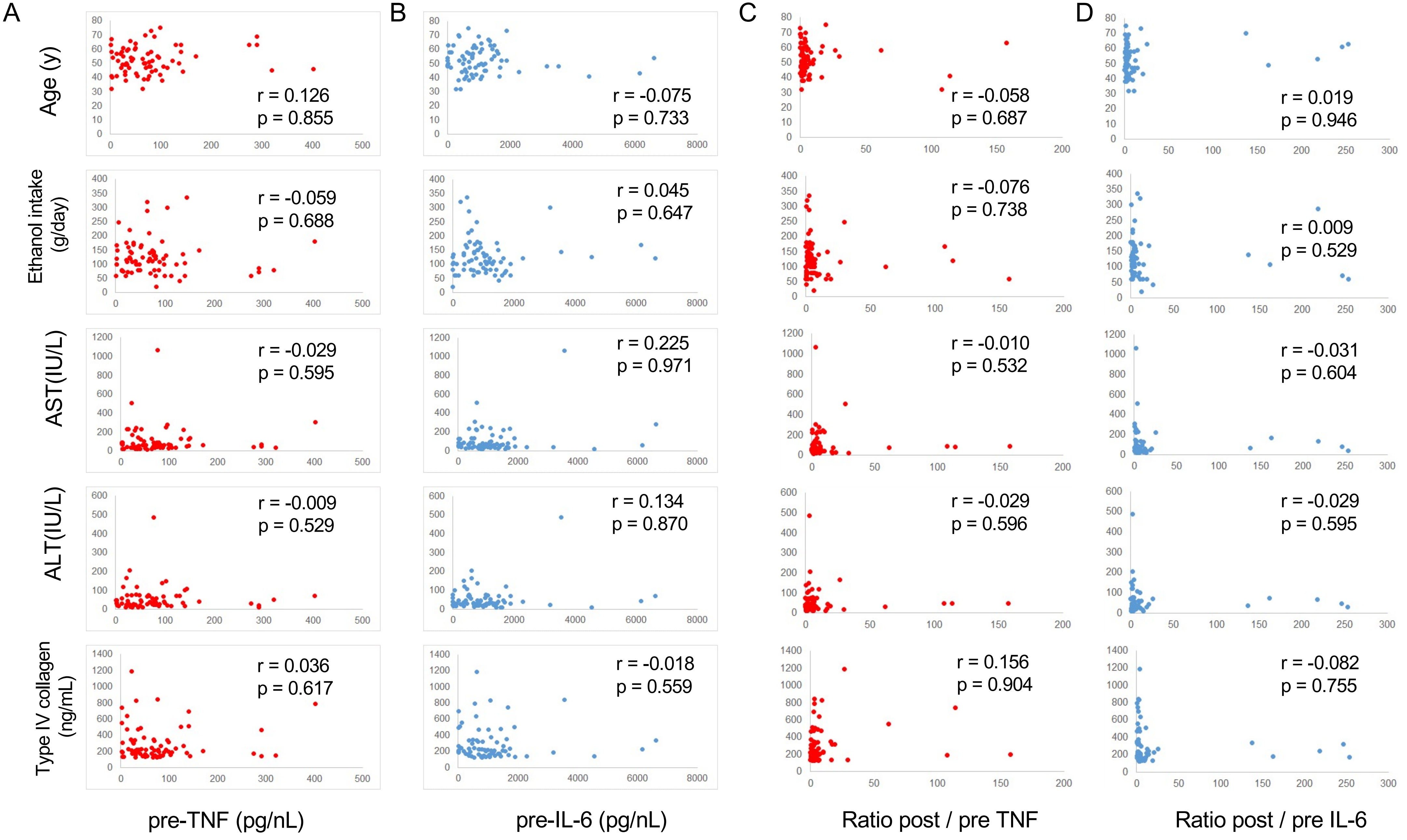

Supplementary Figure 2

Relationship between the amount of TNF (A) or IL-6 (B) production of peripheral CD14⁺ monocytes on admission or the recoveries following alcohol abstinence (TNF; (C), IL-6; (D)) and clinical parameters such as age, ethanol intake, serum AST levels, serum ALT levels, and serum type IV collagen levels.

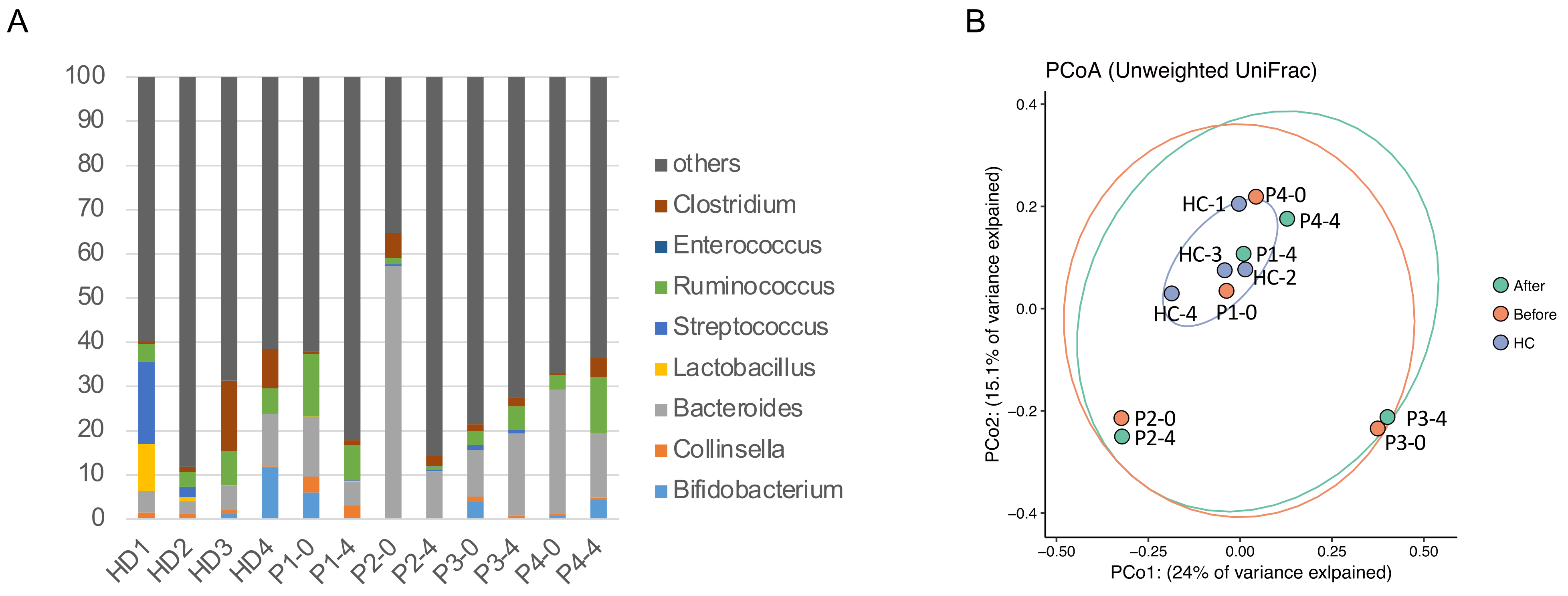

Supplementary Figure 3

Fecal samples were collected from four healthy controls (HCs) and four alcohol dependent patients (AD). The patients' samples were collected at the time of admission (ex; P1-0) and 4 weeks after (ex; P1-4). (A) Composition of microbiota of each sample at the genus level. (B) Principal-coordinate analysis based on the unweighted UniFrac analysis of bacterial-community structures of of each sample.

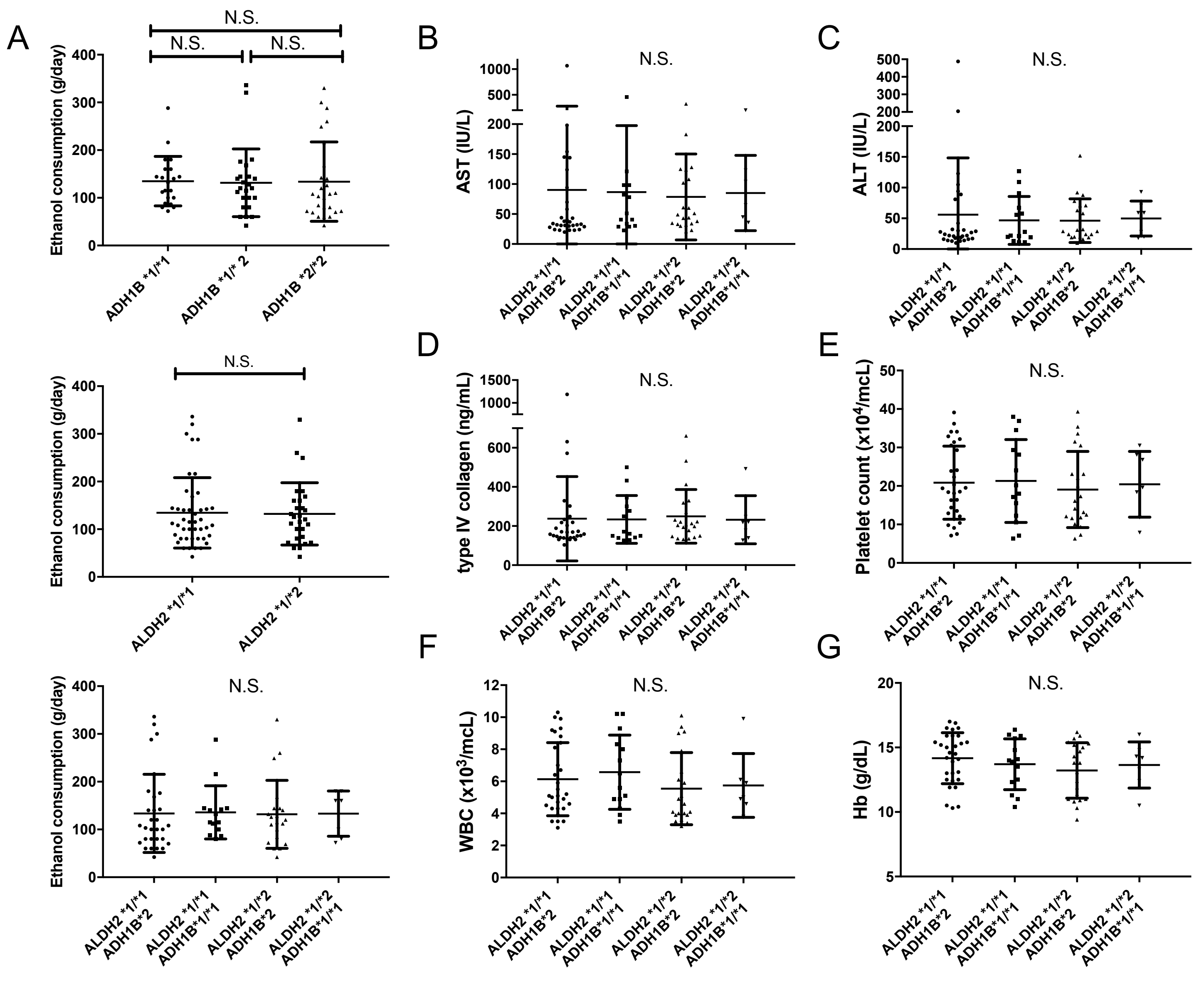

Supplementary Figure 4

(A) Daily dose of ethanol consumption according to the genetic polymorphism in ADH1B (upper), ALDH2 (middle), and their combination (lower). (B) Serum AST levels, (C) serum ALT levels, (D) serum type IV collagen levels, (E) platelet counts, (F) WBC counts, and (G) Hemoglobin (Hb) concentrations of the indicated patients group according to the combination of alcohol-metabolizing enzymes.
